# Cardiac magnetic resonance as the key to uncovering unusual disseminated histoplasmosis: a case report

**DOI:** 10.1093/ehjcr/ytaf408

**Published:** 2025-08-21

**Authors:** Giorgia Benzoni, Ilaria Garofani, Diana Artioli, Cristina Giannattasio, Patrizia Pedrotti

**Affiliations:** Department of Medicine and Surgery, University of Milano-Bicocca, Piazza dell'Ateneo Nuovo, 1, Milan 20126, Italy; Department of Medicine and Surgery, University of Milano-Bicocca, Piazza dell'Ateneo Nuovo, 1, Milan 20126, Italy; Department of Radiology, ASST Grande Ospedale Metropolitano Niguarda, Piazza Ospedale Maggiore 3, Milan 20162, Italy; Department of Medicine and Surgery, University of Milano-Bicocca, Piazza dell'Ateneo Nuovo, 1, Milan 20126, Italy; ‘De Gasperis’ Cardio Center, ASST Grande Ospedale Metropolitano Niguarda, Italy Piazza Ospedale Maggiore 3, Milan 20162, Italy; ‘De Gasperis’ Cardio Center, ASST Grande Ospedale Metropolitano Niguarda, Italy Piazza Ospedale Maggiore 3, Milan 20162, Italy

**Keywords:** Cardiac magnetic resonance, Histoplasmosis, Multimodality imaging, Case report

## Abstract

**Background:**

Disseminated histoplasmosis is a severe fungal infection caused by *Histoplasma capsulatum* which primarily affects immunocompromised individuals, leading to widespread infection in multiple organs such as lungs, liver, and spleen. Early diagnosis and treatment are crucial for effective management.

**Case summary:**

We herein report the case of a 33-year-old male patient who presented to the Emergency Department with fever and chest pain after returning from a journey from a tropical region in Centre America. Initial tests showed elevated high-sensitivity troponin T (Hs-TnT) levels, suggesting possible cardiac involvement, but EKG and chest X-ray were normal. Echocardiography detected hypokinesis of the interventricular septum and a small pericardial effusion. Cardiac magnetic resonance (CMR) showed left ventricular function at lower normal limits and a small pericardial effusion, but also masses in the lungs and mediastinum, confirmed by computed tomography. Biopsy was performed, and histology revealed disseminated histoplasmosis. The patient was treated with antifungals and was discharged after two weeks, continuing antifungal administration in the outpatient clinic for 18 months. Follow-up imaging showed significant reduction of the masses. The patient remained asymptomatic with no further treatment needed.

**Discussion:**

In this case report, we emphasize the essential role of a multimodal imaging approach in diagnosing cardiac inflammatory diseases. CMR was pivotal providing a three-dimensional perspective of the mediastinum, which led to the identification of a retrocardiac mediastinal mass that might have otherwise gone undetected. This highlights the importance of integrating multimodality imaging techniques to improve diagnostic accuracy and guide effective treatment strategies.

Learning points

- Pericardial involvement as a rare feature of disseminated histoplasmosis in an immunocompetent patient highlights the importance of considering fungal infections, even in those with normal immune function.
- The critical role of travel history in patients presenting with myocarditis-like symptoms: this emphasizes the importance of obtaining a detailed travel history in patients with unexplained inflammatory or cardiological symptoms, as travel-related infections can mimic more common cardiovascular conditions.
- The importance of multimodal imaging in diagnosing unexpected conditions: this case highlights the vital role of multimodal imaging—echocardiography, cardiac MRI, and computed tomography—in detecting atypical conditions missed by standard X-rays, enabling early diagnosis and treatment.

## Introduction

Histoplasmosis, caused by inhalation of *Histoplasma capsulatum* conidia, is usually a self-limited infection in immunocompetent individuals.^1^ Disseminated histoplasmosis with multi-organ involvement is rare in healthy patients and more common in immunocompromised hosts.^2^ We report a rare case of disseminated histoplasmosis in a 33-year-old immunocompetent man who developed chest pain and elevated cardiac biomarkers after travel to Central America, where multimodality imaging was crucial for diagnosis.

## Summary figure

**Figure:**
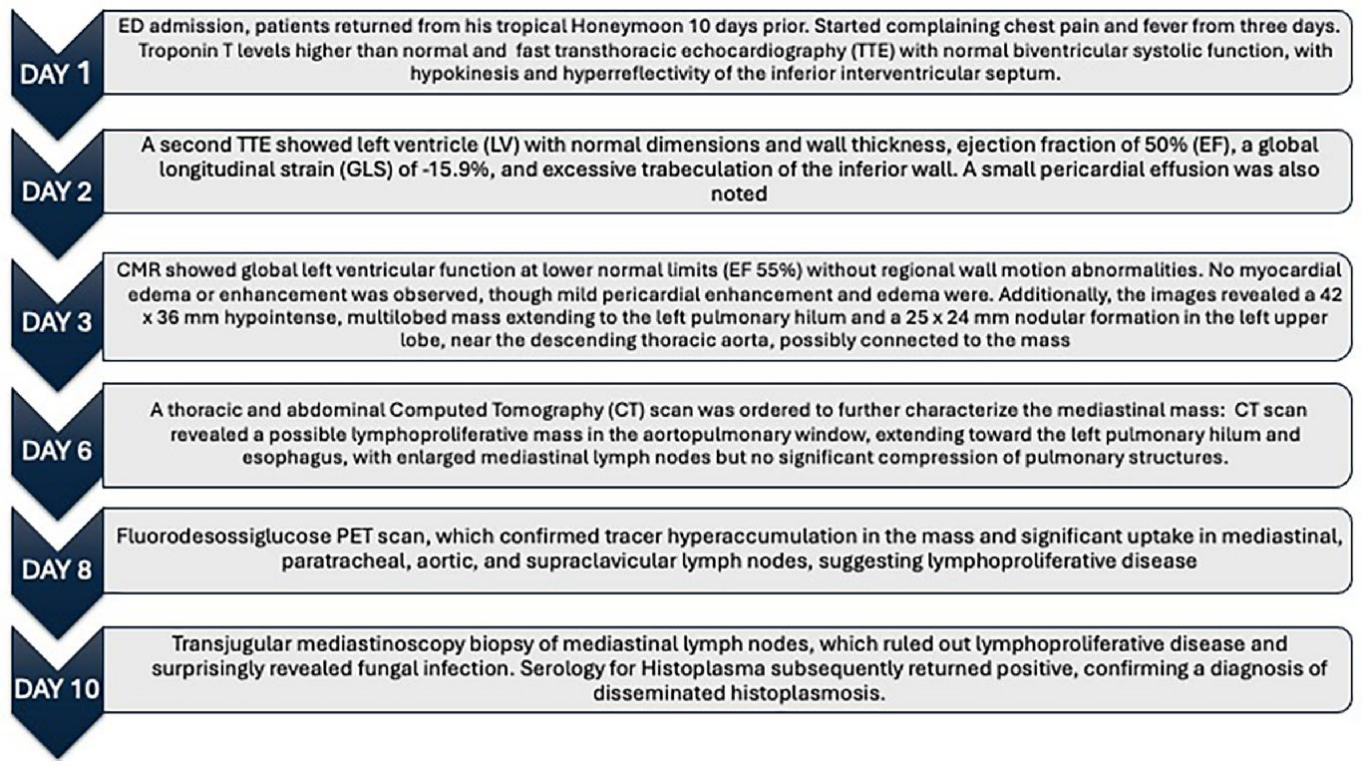


## Case presentation

**Figure 1:**
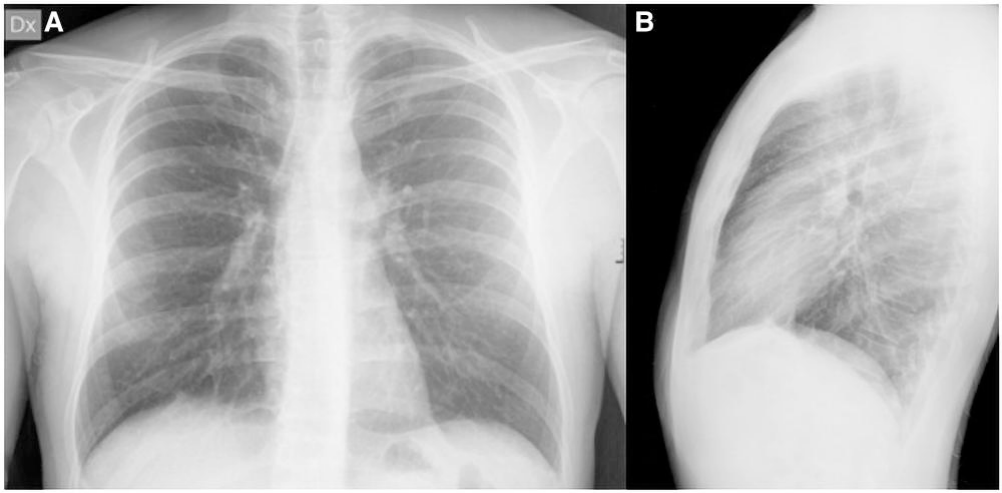
Patient’s ER chest X-ray in antero-posterior (*A*) and latero-lateral view (*B*).

**Figure 2:**
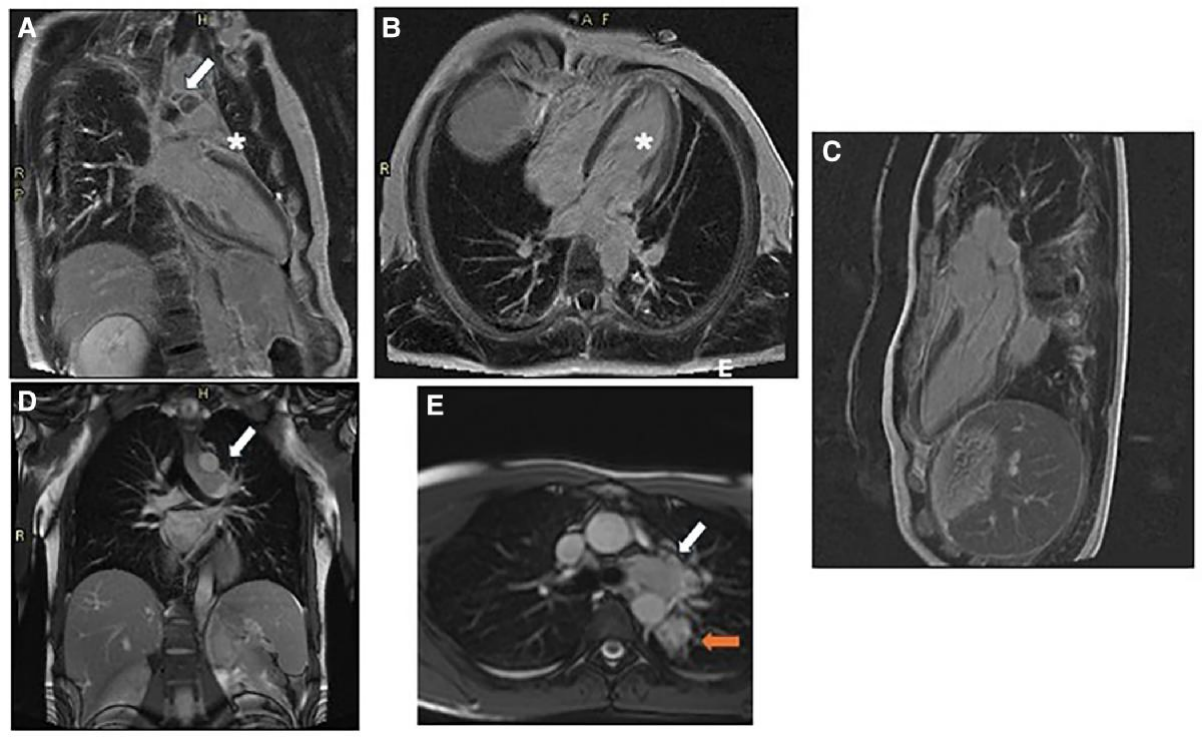
CMR post–contrast sequences (*A–C*) showing mild pericardial enhancement mainly in basal segment of anterior and anterolateral wall (asterisks, *A*, *B*). Localizing images revealing a hypointense, multilobed mass (indicated by upper right arrow, *D*, *E*), and nodular formation in the left upper pulmonary lobe (indicated by lower right arrow, *E*).

**Figure 3:**
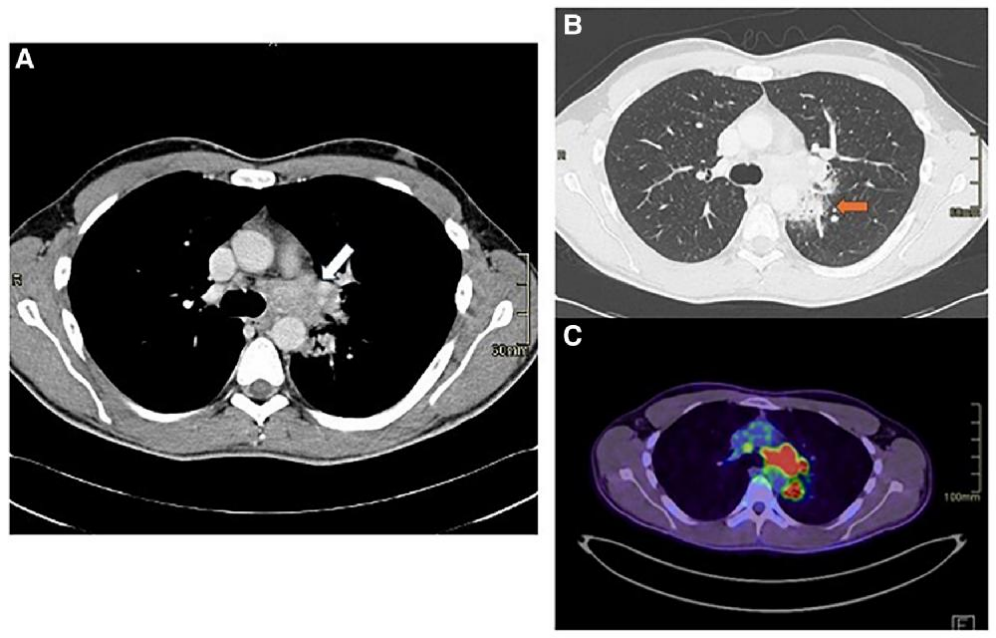
CT scan (*A* and *B*) confirmed the presence of a mass in the aortopulmonary window, extending towards the left pulmonary hilum and oesophagus (indicated by the arrow, *A*) and of a thickening area in the left pulmonary upper lobe (indicated by the arrow, *B*). PET-CT scan showing the tracer hyperaccumulation in the mass and the pulmonary lesion (*D*).

**Figure 4:**
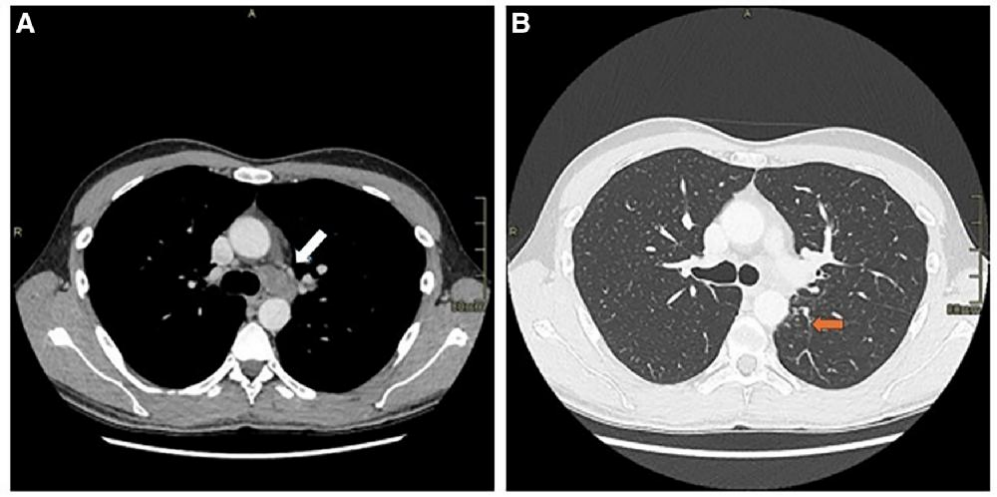
CT scan after 1 year showing the reduction of the mass's dimensions (*A*, indicated by the arrow) and the disappearance of the thickening area in the left pulmonary upper lobe (*B*, indicated by the lower right arrow).

**Figure 5:**
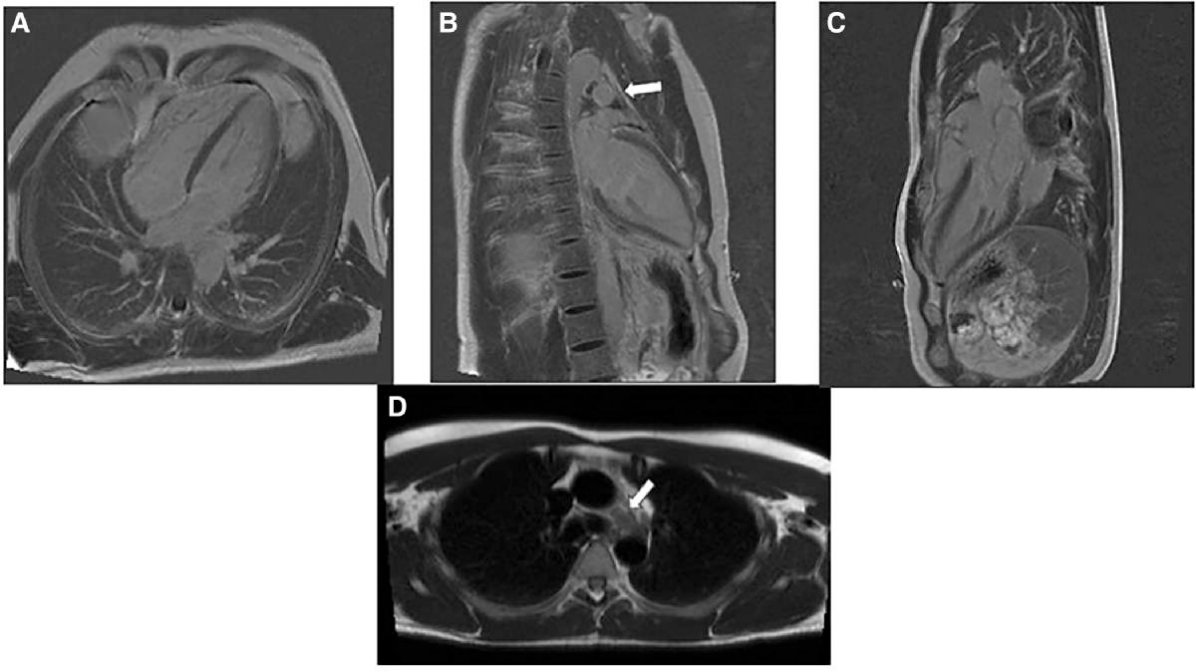
CMR post-contrast sequences after 2 years (*A–C*). Localizing images and post-contrast two-chamber sequence (*B*), revealed a hypointense, multilobed mass (white arrow, *D*).

A 33-year-old man was admitted to our Emergency Department (ED) with fever and chest pain from 3 days, after returning from Centre America. He denied any comorbidities, prior cardiological history or drug abuse. He also denied taking any medication before admission. Upon arrival, the patient was hemodynamically stable, with a blood pressure of 140/75 mmHg, heart rate of 95 b.p.m., and body temperature of 37°C, with peripheral oxygenation of 98% in air. The EKG showed sinus rhythm, without any atrioventricular or intraventricular conduction defects and with normal repolarization (see Supplementary material online, *Figure S1*); chest X-ray did not show any pathological findings (*Figure 1*). Plasmodium infection was excluded through serological test, and Sars COVID-19 nasopharyngeal swab resulted negative. Emergency blood tests revealed elevated troponin T at 50.8 ng/mL (normal range [nr] 0–14 ng/mL), creatinine 1.13 mg/dL (nr 0.67–1.17 mg/dL), and C-reactive protein 2.9 mg/dL (nr 0–0.5 mg/dL), with no significant changes in the leucocytes count. Haemoglobin was 13.7 g/dL (nr 14–18 g/dL), platelets were 190 × 10^9/L (nr 140–440), and NT-proBNP (N-terminal pro B-type natriuretic peptide) was 334 ng/L (nr 0–86 ng/L). Fast transthoracic echocardiography (TTE) revealed normal biventricular systolic function, with hypokinesis and hyperreflectivity of the inferior interventricular septum. No pericardial effusion or valvular abnormalities were observed. The patient was admitted to the Cardiology Department with the suspicion of acute myocarditis. Serology for viruses potentially responsible for myocarditis resulted negative both for acute and recent infections as well as a complete autoimmunity panel. Tests for *Leishmania donovani*, *Brucella*, Zika virus, QuantiFERON, and HIV were also negative. Two sets of blood cultures were collected, which remained negative after 120 h. Troponin and C-reactive protein levels remained stable after 24 h (troponin T 50.5 ng/mL and C-reactive protein 1.5 mg/dL), although the patient experienced further episodes of fever, reaching 38°C. The following days, troponin levels dropped to 10 ng/dL. Immunohematology consultation ruled out immunocompromising diseases, with normal white blood cell counts, serum protein electrophoresis, immunoglobulin levels (A, M, G), lactate dehydrogenase, and β2-microglobulin. TTE 1 day after admission showed left ventricle (LV) with normal dimensions and wall thickness, with mildly reduced ejection fraction (EF) at 50% and global longitudinal strain (GLS) at −15.9%; a small pericardial effusion was also noted; there was no significant valvular disease. Considering pericardial findings, colchicine (1 mg per day) was started, cardiac magnetic resonance (CMR) was performed on a 1.5 T clinical scanner (Magnetom Aera, Siemens Healthineers). The protocol included SSFP (steady-state free precession) cine images on long axis (LA) planes and a short axis (SA) stack from the atrioventricular plane to the apex of the heart. STIR (short-tau inversion recovery) T2-weighted images and PSIR (phase sensitive inversion recovery) post-contrast (gadobutrol, Bayer Schering Pharma©, Berlin, Germany; 0.15 mmol/kg) images were acquired on planes matching all the LA and SA cine images. Parametric maps were acquired on three SA slices, before (T1 native and T2 mapping) and 15 min after contrast administration (post-contrast T1 mapping). The scan revealed normal LV volumes, with normal regional wall motion and EF at lower normal limits (55%). No myocardial oedema or enhancement was observed in any of the myocardial segments; T1, T2 mapping, and ECV calculated in the mid-septal region were within published referenced values. A small pericardial effusion with mild pericardial oedema and enhancement were noted (*Figure 2A–C*). Additionally, localizing images revealed a mediastinal 42 × 36 mm hypointense, multilobed mass extending to the left pulmonary hilum and a 25 × 24 mm nodular formation in the left upper pulmonary lobe, near the descending thoracic aorta, possibly connected to the mass (*Figure 2D* and *E*). A thoracic and abdominal computed tomography (CT) scan was ordered to further characterize the mediastinal mass (*Figure 3A* and *B*), identifying a mass in the aortopulmonary window, extending towards the left pulmonary hilum and oesophagus, with enlarged mediastinal lymph nodes but no significant compression of pulmonary structures. The lesion was unilateral, solid, and composed of confluent nodules, without necrosis or infiltration of vessels and bronchi. The initial diagnosis favoured a lymphoproliferative disease.^3,4^ Lung cancer, sarcoidosis, and tuberculosis (TB) were unlikely due to the absence of aggressive features, bilateral involvement, and necrosis. PET scan confirmed tracer hyperaccumulation in the mass (*Figure 3C*) and significant uptake in mediastinal, paratracheal, aortic, and supraclavicular lymph nodes, suggesting lymphoproliferative disease. The patient underwent *trans* jugular mediastinoscopy biopsy of mediastinal lymph nodes, which ruled out lymphoproliferative disease and surprisingly revealed widespread granulomatous inflammation with extensive necrosis but no giant cells. Grocott staining identified oval fungal structures, suggesting a possible fungal infection. Serology for *Histoplasma* subsequently resulted positive, confirming a diagnosis of disseminated histoplasmosis. The target treatment with intravenous antifungal therapy with liposomal amphotericin B (3 mg/kg for 7 days) followed by itraconazole (600 mg/day) was initiated as soon as the diagnosis was confirmed. Colchicine therapy was then discontinued to avoid interaction with itraconazole, corticosteroid was not administered according to ESC guidelines.^5^ The patient tolerated treatment well and was discharged after two weeks with outpatient follow-up in the infectious disease’s clinic. Before discharge, a cardiological evaluation ruled out acute myocarditis, as there were no EKG changes, CMR findings per modified Lake Louise criteria,^6^ or elevated troponin levels. Post-discharge, itraconazole continued for 18 months. A one-year thoracic CT showed a reduced mediastinal mass (*Figure 4*), and a two-year follow-up CMR confirmed normal LV function with no myocardial abnormalities (*Figure 5*). The patient remains asymptomatic.

## Discussion

Histoplasmosis typically affects immunocompromised patients, while immunocompetent individuals are often asymptomatic or mildly symptomatic. However, disseminated disease can occur without obvious immunodeficiency.^1,7,8^ Our patient’s presentation initially suggested a cardiac cause, which delayed consideration of infectious aetiologies. Pericardial involvement is rare but significant in immunocompetent hosts and often underdiagnosed due to non-specific symptoms.^9^ Cardiac MRI revealed a mediastinal mass missed on chest X-ray, leading to a differential including lymphoma, tuberculosis (TB), sarcoidosis, and fungal infections. Lack of systemic ‘B’ symptoms, normal blood counts, negative QuantiFERON, and non-specific PET-CT favoured an infectious cause. TB and sarcoidosis were less likely due to absence of bilateral hilar lymphadenopathy and granulomas.^10^ This case highlights the importance of detailed travel history and supports early use of CT and CMR in atypical cases, despite guidelines not recommending imaging based solely on travel.^11^ Corticosteroids were withheld per ESC guidelines to avoid worsening an undiagnosed fungal infection.^5^ Although antifungal therapy was delayed, the case emphasizes the need for clinical vigilance for atypical fungal infections in immunocompetent patients with potentially relevant travel exposure.^10^

## Conclusion

This case underscores the importance of broad differential diagnoses in patients with non-specific symptoms such as fever, chest pain, and elevated cardiac markers. Multimodal imaging and travel history were key to diagnosing disseminated histoplasmosis and initiating timely treatment, especially in patients returning from endemic areas.

## Lead author biography


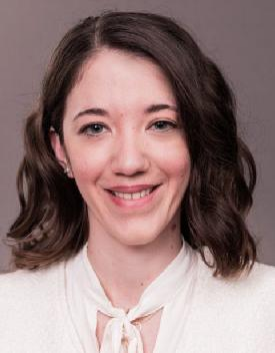


Giorgia Benzoni is a cardiology resident at University of Milan-Bicocca, Italy. During her residency, she developed a strong interest in cardiovascular imaging, with a particular focus on echocardiography and valvular heart disease. She is currently pursuing a fellowship in this field at Ramón y Cajal University Hospital in Madrid, Spain. In addition to her clinical training, she is involved in research projects related to cardiovascular imaging and valvular heart disease.

## Supplementary Material

ytaf408_Supplementary_Data

## Data Availability

The data underlying this article will be shared on reasonable request to the corresponding author.

## Notes

**Consent:** The authors confirm that consent for submission and publication of this case report has been obtained from the patient in line with COPE guidance.

**Funding:** None declared.
