# Supplementary figures and images for: Cardiac magnetic resonance as the key to uncovering unusual disseminated histoplasmosis: a case report
Source: Eur Heart J Case Rep. 2025 Aug 21;9(9):ytaf408. doi: 10.1093/ehjcr/ytaf408 (PMC12418945; doi:10.1093/ehjcr/ytaf408)

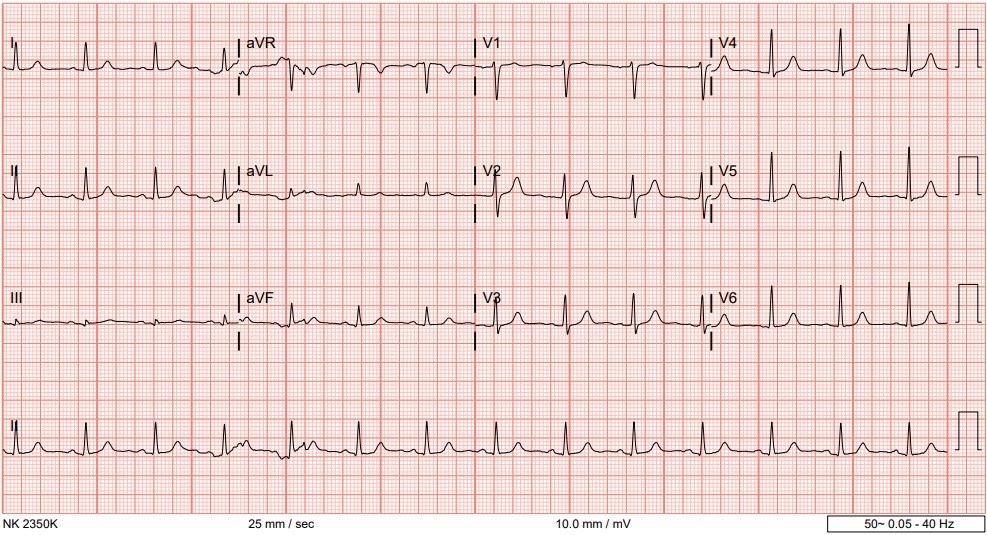

Supplement: ytaf408_Supplementary_Data [file ytaf408_supplementary_data.jpeg]
